# Effect of Age and Body Size on the Wrist's Viscoelasticity in Healthy Participants From 3 to 90 Years Old and Reliability Assessment

**DOI:** 10.3389/fspor.2020.00023

**Published:** 2020-04-07

**Authors:** Anh Phong Nguyen, Benoit Herman, Philippe Mahaudens, Gauthier Everard, Thibaut Libert, Christine Detrembleur

**Affiliations:** ^1^Neuro Musculo Skeletal Lab (NMSK), Institut de Recherche Expérimentale et Clinique, Secteur des Sciences de la Santé, Université catholique de Louvain, Brussels, Belgium; ^2^Institute of Mechanics, Materials and Civil Engineering, Conception, Réalisation et Essais de Dispositifs ElectroMécaniques, Secteur des Sciences technologiques, Université catholique de Louvain, Louvain-la-Neuve, Belgium; ^3^Service d'orthopédie et de traumatologie de l'appareil locomoteur, Cliniques universitaires Saint-Luc, Brussels, Belgium; ^4^Service de médecine physique et réadaptation, Cliniques universitaires Saint-Luc, Brussels, Belgium

**Keywords:** biomechanics, stiffness, elasticity, viscosity, normative data

## Abstract

Excessive or insufficient levels of passive musculoarticular stiffness (PMAS) can lead to joint impairment or instability. Quantifying the PMAS may provide a better understanding of neurological or musculoskeletal disorders. The aims of the present study were multiple: first, to assess the reliability of quantifying PMAS and to collect normative data on the wrist in healthy participants, and second, to assess the effect of age and body size on PMAS. For this purpose, a total of 458 participants from 3 to 90 years old were analyzed with an electromechanical oscillation device (EOD). Passive sinusoidal movements were induced in a flexion/extension pattern in the participants' wrists, enabling an objective measurement of elastic stiffness (EL) and viscous stiffness (VI). Both the dominant and non-dominant wrists were assessed. Two-way repeated-measures ANOVA revealed a sex differentiation from puberty (12–18 years old) and an increase of EL and VI from childhood to adulthood and a decrease of stiffness at old age. EL and VI values were associated with body size characteristics and age. After body size normalization, EL was no longer influenced by the variables measured. On the other hand, VI remained moderately influenced by age and body size. The current study was able to provide normative data of PMAS in the wrist of healthy participants.

## Introduction

Assessment of passive musculoarticular stiffness (PMAS) is of a growing interest in clinical practice and sport to diagnose, prevent, or treat musculoskeletal injuries. Inadequate levels of PMAS have been associated with the occurrence of movement dysfunction, development of pathologies, and reduction in performance. PMAS can be defined as the ratio between the load applied and the deformation that occurs in a structure (Blackburn et al., 2014); i.e., the greater the PMAS, the less the applied load impacts the deformation. PMAS is often differentiated from active musculoarticular stiffness that is generated by contractile structures of the muscles and reflex pathways (Detrembleur and Plaghki, 2000). For its part, PMAS is due to the rheological properties of non-contractile elements such as muscle–tendon units, aponeurosis, and capsuloligamentous complex (Leger and Milner, 2000; Kuo and Deshpande, 2012). Furthermore, Riemann and Schmitz described that PMAS included the contribution from all the tissues located around and within the joint, such as muscle–tendon units, skin, subcutaneous tissues, facias, ligaments, joint capsule, and cartilage (Riemann and Schmitz, 2012). Those passive components may be influenced by several factors, including anatomical properties of muscular tissues, age, sex, and body size characteristics, but also the surrounding temperature, hour of the assessment, strain rate and amplitude, history of previous pathologies, or immobilization. It is known that men develop higher PMAS than women, but PMAS values become non-significant between sex after body size normalization (Blackburn et al., 2014). Several authors have demonstrated the correlation between body size characteristics and PMAS values, e.g., in the knee or elbow (Chleboun et al., 1997; Blackburn et al., 2004; Dionysian et al., 2005). Ditroilo et al. (2012) showed that when the normalized results were compared between older (65 years) and younger subjects (22 years), no differences were found in PMAS. Kubo et al. (2001) observed that the tendon structures were more compliant in young boys (11 years) than those in older boys (15 years) and young men (25 years). However, no studies have described the relationship between all ages of life and PMAS.

PMAS has already been objectively and quantitatively measured distally by inducing sinusoidal oscillations, as demonstrated by Rack (1966) or Lehmann (1989) and adapted by Detrembleur and Plaghki (2000). The distal unit, like the ankle or wrist joint, can be simplified in a mechanical model like a torsional spring, a viscous torsional damper and a rotatory mass. These torsional elements seem appropriate as the system is being rotated around the joint. The electromechanical oscillation device (EOD) can measure the resistance of the joint under passive sinusoidal displacements at different frequencies of oscillation. EOD can provide a characteristic torque response, which is dependent on the particular mechanical properties of the model's elements (Detrembleur and Plaghki, 2000). Torsional spring will correspond to a torque response in phase with the displacement, but independent of the frequency, while the torsional viscous damper is related to the frequency of the sinusoidal displacement. Torque contribution of the mass can be computed and subtracted from the total response and provides net PMAS, composed of elastic stiffness and viscous stiffness (Detrembleur and Plaghki, 2000). This quantitative device can, therefore, assess the PMAS of a distal joint such as the ankles, fingers, or wrists (Dierick et al., 2011).

The primary aim of the current study was to investigate the intra- and inter-rater reliability of the EOD in healthy participants. Our secondary aim was to observe the relationship between age and body size characteristics on the wrist's PMAS in a healthy population (in 458 participants aged from 3 to 90 years old). To our knowledge, no normalized data of PMAS in the wrist have been acquired thus far. This study could offer such data on PMAS and provide a better understanding concerning the assessment and treatment of musculoskeletal disorders or neurological pathologies such as forearm spasticity, tremor in Parkinson's disease, wrist arthritis, or inflammation.

## Materials and Methods

### Population

The study was performed between July 2018 and December 2018 in Université Catholique de Louvain (UCLouvain) in Brussels and Louvain-la-Neuve, Belgium. Healthy participants of both sex aged between 3 and 90 years old were recruited by word of mouth; advertisements in local schools, at the university, and surroundings; or investigators' acquaintances. Four hundred fifty-eight participants were enrolled in the current study. Seven were removed due to technical problems during the data collection. Among the 451 remaining participants, 148 children (72 girls and 76 boys) and 303 adults (162 women and 141 men) constituted our sample (see Table 1). Childhood was defined by chronological age. No participants reported any pain or inconvenience during or after the data collection. To participate in the study, the participant had to meet the inclusion criteria, i.e., no history of injury or pathology in the upper limb and having no pain or any condition that might cause discomfort during the study. The current study was approved by the local ethics committee: “Comité d'éthique Hospitalo-Facultaire de l'UCLouvain” (CEHF) (Belgian registration number: B403201523492) in March 2017. All participants and parents of children were informed about the protocol before any measurement and signed written informed consent.

**Table 1:** Reliability of EOD in wrist's stiffness.

		**Reliability**	**Standard error of mean**		**Responsiveness**	
	**Mean** **±** **SD**	**ICC (95% CI)**	**SEM**	**SEM%**	**MDC**	**MDC%**
**INTRA-EXAMINER**						
Elastic stiffness (N m rad^−1^)	1.716 ± 0.64	0.878 (0.836–0.91)	0.223	34.9	0.619	36.1
Viscous stiffness (N m s rad^−1^)	1.922 ± 0.68	0.931 (0.907–0.949)	0.181	26.3	0.500	26.0
**INTER-EXAMINER**						
Elastic stiffness (N m rad^−1^)	1.742 ± 0.60	0.941 (0.909–0.961)	0.154	24.3	0.428	25.0
Viscous stiffness (N m s rad^−1^)	1.966 ± 0.67	0.931 (0.895–0.955)	0.178	26.3	0.495	25.9

*ICC, intraclass correlation coefficient; SEM, Standard error of mean; MDC, minimal detectable change*.

### Body Size Measurements

For all participants, age, height, body mass, forearm length (from the head of the radius to radial styloid process with a ruler) and forearm circumference (at the largest part of the forearm) were recorded. Wrist range of motion was measured with a goniometer in full flexion and full extension. All the measurements were taken for both sides while the participants sat with the forearm on the examination table. The dominant side was defined as the writing hand of the participant.

### Electromechanical Oscillation Device

**Figure 1:**
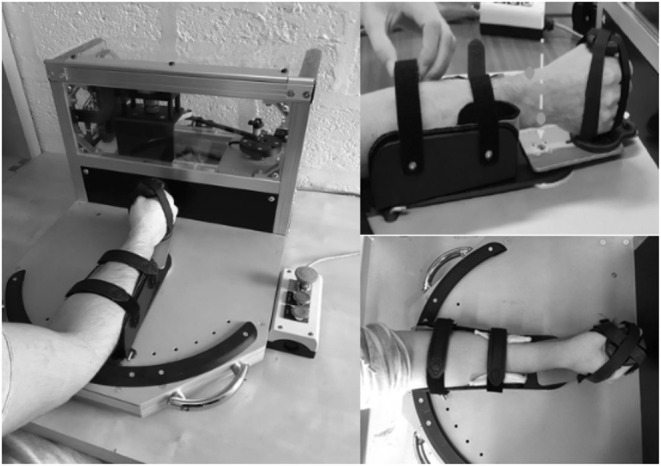
Electromechanical oscillation device adapted by Detrembleur and Herman. The arrow shows the rotatory axis. The dot represents the radial and ulnar styloid process of the forearm.

Inspired by the work of Rack (1966) and Lehmann (1989) and adapted by Detrembleur and Plaghki (2000) in the ankle, we developed in collaboration with the Institute of Mechanics, Materials and Civil Engineering of the University of Louvain (UCLouvain), an electromechanical device applying sinusoidal rotatory movements of varying frequencies to objectively assess the passive wrists' musculoarticular stiffness. We were inspired by existing devices to build one with as little inertia as possible, portable, easy to use in a clinical context, safe, reliable, and reproducible. The participants' wrists were mobilized from 3 to 12 Hz in 1-Hz increment (Lehmann, 1989). The movement was driven by the gripping handle and oscillation movements were performed from 10° of wrist flexion to 10° of wrist extension for a total of the mean amplitude of 20° with a precision of 0.8°. A neutral wrist position at 0° of flexion/extension was chosen (Pando et al., 2014). The pilot protocol showed no significant difference in starting position in extension or flexion. In addition, if needed, a piece of foam was placed into the opencast to limit the movement of the forearm. A torquemeter (Sensy 62200 of 75 Nm—SENSY S.A. Jumet, Belgium) placed on the gripping handle axis measured torsion forces while a potentiometer (Codeur SICK absolute 4096 p/t- SICK NV/SA Asse, Belgium) mounted on the axis of rotation recorded angular displacement. The forearm was fixed by a strap in an opencast and the hand was stabilized by four straps around the hand and fingers (Figure 1).

**Figure 2:**
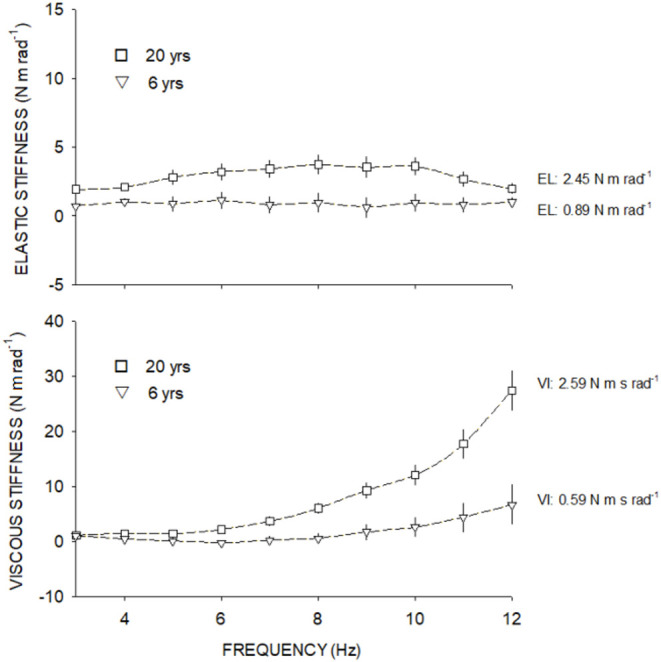
Elastic stiffness (above) and viscous stiffness (below) at each frequency from 3 to 12 Hz. Square symbols show the elastic and viscous stiffness from a 20-year-old adult and triangle symbols show the elastic and viscous stiffness of a 6-year-old child.

Participants ideally sat with the forearm relaxed, and the elbow flexed between 120° and 135° and the shoulder at 45° of flexion. Angle and torque were recorded in 1024 Hz and analyzed by a fast Fourier transformation (FFT). Only the first harmonic, corresponding to the gripping handle oscillation frequency, was preserved. The amplitude and phase shift of the torque signal relative to the displacement signal were computed. The amplitude of the torque response that is in phase with the wrist displacement is called the elastic torque and the one that is 90° out of phase with the displacement is called the viscous torque. The influence of combined gripping handle and hand inertia (*I*) on the amplitude of the elastic torque and device friction on the amplitude of the viscous torque were subtracted, respectively, from raw amplitude values. Inertia was calculated as the slope of the regression line from total elastic stiffness values vs. frequency squared. The net elastic stiffness at each frame was obtained by subtracting inertia from total stiffness. The amplitude of the elastic (EL) and viscous (VI) torque components was expressed in terms of stiffness (N m rad^−1^) and computed as the ratio of net torque amplitude (raw torque amplitude without the effects of inertia or friction) to wrist displacement. EL and VI results were expressed as the mean computed from the three measures recorded at each frequency. Elastic stiffness was represented by the intercept of the regression line fitted through points (elastic stiffness vs. frequency) and expressed in N m rad^−1^. Viscous stiffness was represented by the slope of the regression line fitted through points (viscous stiffness vs. frequency) and expressed in N m s rad^−1^ (Figure 2).

### Experimental Procedure

#### Reliability Study

Intra and inter-reliability were tested by two operators assessing each participant twice in one session (T_1_-T_2_-T_3_-T_4_). All measurements were performed on the dominant limb, defined as the writing hand of the participants. The total duration of one assessment lasted 195 s, with 1 min of rest between each assessment.

#### PMAS Study

Participants were assessed twice in one session, one assessment per wrist. Each assessment consisted of 30 trials of 10 different frequencies of oscillation (3–12 Hz). The order of frequencies was randomized to avoid pattern prediction.

### Statistical Analysis

#### Reliability Study

Data were analyzed using SPSS 16.0. A two-way random intraclass correlation coefficient (ICC 2,1) was used to assess interoperator reliability, and a two-way mixed ICC (3,1) was used to calculate intraoperator reliability. Following the Shrout and Fleiss classification, ICCs > 0.75 were considered excellent, those between 0.4 and 0.75 were moderate, and those <0.4 were weak (Shrout and Fleiss, 1979). Standard errors of the mean (SEM) were calculated using Equation (1) (Weir, 2005):

(1) $$\begin{array}{l} \text{SEM}=\text{S}{\text{D}}_{\text{x}}\sqrt{1-R_{\text{x}}} \end{array}$$

where SD represents the standard deviation, *x* represents the session, and *R* represents the ICC (3,1). The minimal detectable change (MDC) was then calculated as follows (Equation 2):

(2) $$\begin{array}{l} \text{MDC}=1.96\;\sqrt{2\text{ SEM}} \end{array}$$

where 1.96 is the two-sided table *z* value for the 95% CI and √2 is used for the variance of two measurements (Weir, 2005). SEM and MDC were also expressed as percentages.

#### PMAS Study

Statistical analysis was performed by SigmaPlot version 14 software. Wilcoxon signed-rank test (as normality test failed) was used to establish dominance effect on PMAS in all participants.

To eliminate the body size effect, EL and VI values were also normalized following Hof (1996) equation [Equations (3) and (4)].

(3) $$\begin{array}{l} \text{Normalized EL}=\frac{\text{EL}}{\text{Body mass}\times g\times \frac{\text{Forearm length}}{\text{Height}}}\times \alpha \end{array}$$

where EL represents elastic stiffness values, *g* represents gravitational acceleration, and α denotes the angular acceleration.

(4) $$\begin{array}{l} \text{Normalized VI}=\frac{\text{VI}}{\text{Body mass }\times \;g\;\times \;\frac{\text{Forearm length}}{\text{Height}}\times \;\omega }\;\times \;\alpha^{2} \end{array}$$

where VI represents de Viscous stiffness values, *g* denotes the gravitational acceleration, ω represents the angular velocity, and α denotes the angular acceleration.

Next, seven groups of ages were determined: 3–6, 6–12, 12–18, 18–25, 25–45, 45–65, and 65–90 years old for the following statistical analysis. Descriptive analysis was used on the dominant limb and results are expressed as mean ± SD. Two-way ANOVA was used to establish the effect of age and sex on stiffness.

To characterize the association between age, height, body mass, sex, forearm circumference, forearm length, ROM flexion, ROM extension (*X*_i_ variables), and stiffness variables (*Y*_i_), a polynomial regression (first or second order) was adjusted. The choice of order was verified by the highest r and significant *p*-value. The coefficients *r* were classified as follows: 0–0.25 = very low, 0.26–0.49 = low, 0.5–0.69 = moderate, 0.7–0.89 = high, and 0.9–1.00 = very high. The significance level was fixed at *p* = 0.05.

## Results

### Reliability of EOD

Eighty-six participants (46 female), ranging in age from 5 to 90 years old (29.58 ± 16.8 years), were included in the reliability study. Intraoperator reliability (Table 1) was excellent for all parameters (ICCs ranged from 0.87 to 0.93). Interoperator reliability showed similar results, with an ICC > 0.93 for all stiffness components. The intraoperator SEM values for EL and VI were 0.22 N m rad^−1^ and 0.18 N m s rad^−1^, respectively. Interoperator SEM values were lower than intraoperator values for ES (0.14 N m rad^−1^) and VI (0.17 N m s rad^−1^). All the MDC values are shown in Table 1.

### PMAS and Wrist Dominance

No significant difference was observed between dominant and non-dominant wrist in EL [*p* = 0.069; Dominant (Median; 1st quartile to 3rd quartile) = 1.33 (0.85–1.96) N m rad^−1^; Non-dominant = 1.30 (0.84–1.89) N m rad^−1^] and VI [*p* = 0.47; Dominant (Median; 1st quartile to 3rd quartile) = 1.57 (0.77–2.23) N m s rad^−1^; Non-dominant = 1.59 (0.90–2.11) N m s rad^−1^]. As dominance showed no significant interest, further analysis was only made on the dominant side.

### PMAS and Sex Difference

Two-way ANOVA showed a significant difference between sex and age in both EL and VI components. Sex difference only appeared after the 12- to 18-year-old group, while the 3- to 6-year-olds (*p* = 0.893) and 6- to 12-year-olds (*p* = 0.951) showed no significant differences between males and females concerning EL and VI values. The 12–18, 18–25, 25–45, 45–65, and 65- to 90-year-old groups showed higher EL and VI values in males than females (see Table 2).

**Table 2:** Descriptive statistics on body size variables and stiffness.

**Age group (years)**	**Sex**	***n***	**Height (m)**	**Body mass (kg)**	**Forearm circumf. (m)**	**Forearm length (m)**	**ROM Flex (^**°**^)**	**ROM Ext (^**°**^)**	**EL** **(N m rad^**−1**^)**	**VI** **(N m s rad^**−1**^)**
3–6	F	16	1.06 ± 0.08	18.05 ± 3.45	0.17 ± 0.01	0.16 ± 0.01	107.63 ± 6.81	93.81 ± 5.31	0.41 ± 0.13	0.13 ± 0.14
	M	23	1.07 ± 0.08	17.49 ± 3.35	0.18 ± 0.01	0.16 ± 0.02	108.3 ± 12.41	92.44 ± 6.42	0.45 ± 0.23	0.16 ± 0.15
6–12	F	22	1.37 ± 0.12	32.48 ± 7.49	0.2 ± 0.02	0.21 ± 0.02	107.59 ± 10.9	90.73 ± 4.65	0.69 ± 0.17	0.67 ± 0.27
	M	14	1.38 ± 0.12	33.19 ± 7.48	0.21 ± 0.02	0.22 ± 0.02	102.5 ± 9.35	90.43 ± 11.09	0.71 ± 0.25	0.66 ± 0.35
12–18*	F	34	1.65 ± 0.07	53.53 ± 7.53	0.25 ± 0.01	0.27 ± 0.02	87.06 ± 3.22	83.29 ± 5.39	1.43 ± 0.33	1.7 ± 0.39
	M	39	1.72 ± 0.09	58.6 ± 11.67	0.26 ± 0.02	0.29 ± 0.02	86.08 ± 4.72	80.08 ± 8.35	1.75 ± 0.48	1.99 ± 0.57
18–25*	F	44	1.68 ± 0.06	61.85 ± 9.34	0.24 ± 0.02	0.25 ± 0.02	88.07 ± 7.82	67.25 ± 12.17	1.4 ± 0.41	1.64 ± 0.45
	M	50	1.81 ± 0.06	75.19 ± 10.98	0.28 ± 0.02	0.28 ± 0.02	89.16 ± 9.83	72 ± 9.39	2.07 ± 0.35	2.41 ± 0.41
25–45*	F	37	1.68 ± 0.06	66.24 ± 12.10	0.26 ± 0.02	0.23 ± 0.02	89.89 ± 9.87	68.27 ± 15.03	1.64 ± 0.49	1.89 ± 0.45
	M	41	1.80 ± 0.07	79.36 ± 11.76	0.28 ± 0.02	0.26 ± 0.03	85.29 ± 9.59	68.19 ± 12.99	2.22 ± 0.44	2.56 ± 0.43
45–65*	F	38	1.68 ± 0.07	72.01 ± 13.58	0.25 ± 0.02	0.26 ± 0.01	93.37 ± 11.26	87.76 ± 16.26	1.69 ± 0.43	1.92 ± 0.49
	M	33	1.8 ± 0.07	87.94 ± 11.02	0.29 ± 0.02	0.29 ± 0.02	89.24 ± 14.74	85.49 ± 13.07	2.25 ± 0.51	2.45 ± 0.51
65–90*	F	44	1.61 ± 0.06	61.61 ± 11.67	0.24 ± 0.02	0.25 ± 0.02	63.29 ± 10.28	65.46 ± 11.19	1.09 ± 0.43	0.97 ± 0.43
	M	18	1.69 ± 0.07	70.17 ± 13.82	0.26 ± 0.02	0.26 ± 0.02	72.33 ± 14.16	63.89 ± 12.43	1.65 ± 0.83	1.59 ± 0.84

Results are provided in (mean ± SD). circumf, circumference; ROM, range of motion; flex, flexion; Ext, extension; EL, elastic stiffness; VI, viscous stiffness; F, female; M, male; Na, missing values. *Indicated a significant difference between male and female for EL and VI values.

### PMAS and Aging

**Figure 3:**
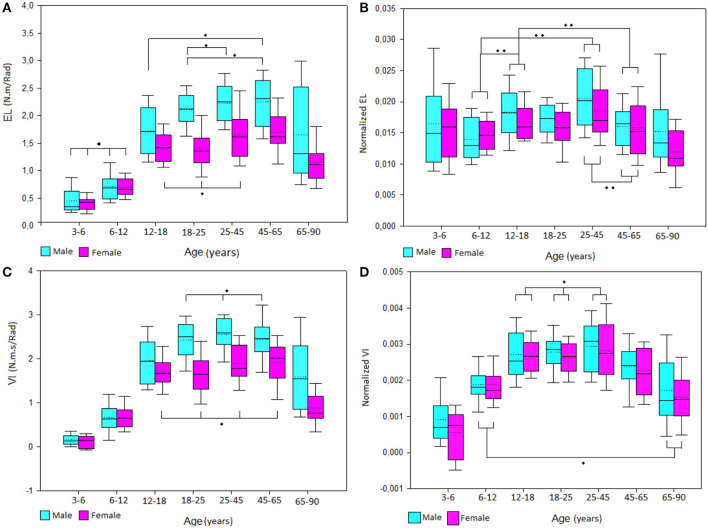
Boxplot of male and female **(A)** elastic stiffness (EL) and **(C)** viscous stiffness (VI) values and normalized **(B)** EL and **(D)** VI values by group of age. *No significant differences between groups, **Significant differences between group.

The results showed an increase of EL stiffness during the lifetime with a similar strategy between males and females with the male group increasing further after the 12- to 18-year-old group and both male and female values dropping after 65 years old (Figure 3). Normalized EL values for both male and female showed an equalizing of values from the 3- to 6-year-old group to the 65- to 90-year-old group (Figure 3).

VI stiffness showed a similar pattern than EL with an increase of values from childhood to adulthood and a decrease of values after the 45- to 65-year-old group. However, normalized VI values kept the same evolution after removing the height, body mass, and forearm length influences. Also, normalized VI values did not show any sex differences (*p* = 0.056). All age group relationships are illustrated in Figure 3.

### PMAS and Body Size Characteristics

Polynomial regressions (first or second order) were adjusted between normalized and not normalized stiffness and body size characteristics (Table 3).

**Table 3:** Polynomial regression (first order: *Y*_i_ = *Y*_0_ + *b*_0_· *X*_i_ or second order: *Y*_i_ = *Y*_0_ + *b*_0_· *X*_i_ + *b*_1_· *X*_i_^2^) on elastic and viscous stiffness variables (*Y*_i_) (not and normalized).

	**Order**	**Equation**	***r*^2^**	***r***
**EL**				
Age (years)	2	0.445 + (0.0688 * Age) – (0.000706 * Age^2^)	**0.42**	**0.65**
Sex (1 = woman)	1	0.82 + (0.479 * Sex)	0.12	0.35
Height (m)	1	−2.407 + (2.411 * Height)	0.57	0.75
Body mass (kg)	1	0.0335 + (0.0244 * Body mass)	0.57	0.75
Forearm circumference (m)	1	−2.030 + (14.273 * circumference)	0.58	0.76
Forearm length	1	−1.390 + (11.672 * Forearm length)	**0.46**	**0.68**
ROM flexion (°)	2	−3.546 + (0.130 * ROM flex) – (0.000794 * ROM flex^2^)	**0.19**	**0.44**
ROM extension (°)	1	2.183 – (0.00848 * ROM ext)	0.04	0.20
**VI**				
Age (years)	2	0.324 + (0.0888 * Age) – (0.000938 * Age^2^)	**0.47**	**0.69**
Sex (1 = woman)	1	0.87 + (0.536 * Sex)	0.09	0.30
Height (m)	1	−3.513 + (3.181 * Height)	0.71	0.84
Body mass (kg)	1	−0.189 + (0.0304 * Body mass)	0.59	0.77
Forearm circumference (m)	1	−2.847 + (18.143 * circumference)	0.63	0.79
Forearm length	1	−2.136 + (15.249 * Forearm length)	0.53	0.73
ROM flexion (°)	2	−5.509 + (0.180 * ROM flex) – (0.00109 * ROM flex^2^)	**0.19**	**0.44**
ROM extension (°)	1	2.507 – (0.0108 * ROM ext)	0.03	0.17
**NORMALIZED EL**				
Age (years)	2	0.0150 + (0.000168 * Age) – (0.00000232 * Age^2^)	0.08	0.28
Sex (1 = woman)	1	0.013 + (0.002 * Sex)	0.05	0.22
Forearm circumference (m)	1	0.0126 + (0.0161 * Forearm circumference)	0.01	0.10
ROM flexion (°)	1	0.0136 + (0.0000335 * ROM flex)	0.01	0.10
ROM extension (°)	1	0.0171 – (0.00000721 * ROM ext)	0.00	0.00
**NORMALIZED VI**				
Age (years)	2	0.00132 + (0.0000744 * Age) – (0.000000853 * Age^2^)	**0.47**	**0.69**
Sex (1 = woman)	1	0.002 + (0.0003 * Sex)	0.03	0.17
Forearm circumference (m)	1	−0.000749 + (0.0124 * Forearm circumference)	**0.24**	**0.49**
ROM flexion (°)	2	−0.006 + (0.0002 * ROM Fl) – (0.000001 * ROM fl^2^)	**0.19**	**0.44**
ROM extension (°)	1	0.00324 – (0.0000118 * ROM ext)	0.03	0.17

*X_i_ are independent variables characterizing body size effect and age. EL, elastic stiffness; VI, viscous stiffness; ROM, range of motion; bold, moderation association; underline, good association; r^2^, determination coefficient; r, correlation coefficient*.

EL was highly associated to height, body mass, and forearm circumference. It was moderately correlated to age, forearm length, and ROM flexion. VI was highly associated to height, body mass forearm circumference, and forearm length. It was moderately associated to age and ROM flexion. After normalization, EL was no longer influenced by age or body size. However, normalized VI remained moderately associated to age, forearm circumference, and ROM flexion (Figure 3).

## Discussion

Our results showed normative data of PMAS in 451 healthy participants ranging from 3 to 90 years old. EOD showed excellent intra- and interoperator reliability in a healthy population between 5 and 90 years old. Our results also suggest that body size characteristics such as height, body mass, and forearm circumference are the most influencing factors for EL and VI components. After eliminating the effect of body size, we observed that aging seems to impact VI rather than EL.

### Reliability

Our results showed excellent intra- and interoperator reliability of the EOD in healthy participants of 5 to 90 years old. Lobet et al. (2018) showed comparable results in their study on the ankle, with good-to-excellent intra- and interday reliability in PMAS of the ankle in children, adolescents, and adults with hemophilia using the same assessment protocol. The reliability study showed that EOD might not be as accurate with the standard error of the mean (SEM) around 10% and MDC of 25%; this might be related to excessive forearm movement into the cast during the acquisitions at high frequencies or non-linear soft tissue behavior at high frequencies and should continue to be investigated.

### PMAS and Dominance

Our study reported no difference in PMAS between the dominant and non-dominant wrist. Several publications showed higher passive stiffness in the dominant upper limb (Clerke and Clerke, 2001; Ingalls, 2004; Durand et al., 2019). Durand et al. (2019) recently described the influence of handedness in passive wrist stiffness, suggesting that differences between studies were probably caused by sample size and age of participants. However, EOD only assessed the flexion/extension axis while Durand et al. (2019) assessed flexion/extension and ulnar/radial axis. Moreover, the velocity of our assessment was greater than in the previously cited study. Dominant and non-dominant limbs could display different properties following their daily activities. Some authors also described the relationship between the development of intramuscular fibers, i.e., type I or type II, and the intensity of physical activities. They explained that repetitive movement in one limb induced an oxidative adaptation of type I fibers without modifying their volume while loaded activities induced the hypertrophy of both the type I and II (Adam et al., 2017). Therefore, sports or professional activities could have an influence on the morphological properties of the participants. More accurate assessment of specific asymmetric populations (i.e., tennis players, musicians, carpenters, laborers, etc.) may show different results. No significant PMAS difference was observed for the dominant and non-dominant wrist. However, adults might demonstrate asymmetrical PMAS between wrists with dominant PMAS higher than non-dominant wrist.

### PMAS and Sex

First, children demonstrated significantly weaker PMAS than adolescents or adults. Furthermore, men showed significantly higher PMAS than women. These results are in concordance with the literature (Kubo et al., 2002; Blackburn et al., 2006, 2014). Body size characteristics do not differ between boys and girls before puberty. Sexual dimorphism is induced by sex steroid hormones during puberty (Wells, 2007). In accordance with this theory, sex differences were apparent toward the 12- to 18-year-old group in the current study. Previous research demonstrated that male participants displayed more stiffness than female participants, and it could be due to a greater height and body mass (Blackburn et al., 2004, 2006, 2014; Durand et al., 2019). Another study pointed out that muscular fiber diameter did not differ in boys and girls before the age of 15 (Oertel, 1988). We showed that height, body mass, or forearm circumference was mostly associated to PMAS components. Similar results were found by Decostre et al. (2015) concerning the wrist strength. Indeed, height and forearm circumference influenced the wrist strength in children while forearm circumference, age, and sex were predictable factors in the adult population (Decostre et al., 2015). Concomitantly, another study confirmed with a hand-held dynamometer that sex, body mass, and age might be predictive factors for wrist extensors' strength (Andrew et al., 1996).

### PMAS and Aging

PMAS is the result of not only muscle rheology (viscoelasticity) but also articular structures such as the joint capsule, ligaments, skin, and fat tissues. It seems thus impossible to isolate the impact of age from the many other factors that vary through the life of a subject (Distefano and Goodpaster, 2018). While height and body mass are associated with both viscoelastic elements, after eliminating the size effect, age and forearm circumference only tend to explain VI, not EL. Indeed, forearm circumference's impact in PMAS may be explained by the number of intramuscular crossbridge (Blackburn et al., 2004, 2006). It is difficult to analyze the isolated impact of one structure in the joint. Diminution of fiber dimensions, intramuscular fat deposit, and insulin resistance are several factors that must be considered in aging and PMAS. Such degenerations could affect the structural organization of the surrounding tissues around the wrist (Makizako et al., 2017). Skin aging can also play a role in PMAS as intrinsic and extrinsic factors induce skin modifications. Several hypotheses, such as oxidative stress, DNA damage, telomere shortening, or inflammaging, could influence the skin aging process (Zhang and Duan, 2018). Distribution of collagen, elastin, or proteoglycans in the extracellular matrix can affect the tensile strength, elasticity, and hydration of the aged skin (Mora Huertas et al., 2016). Wilke et al. (2019) described an age-related modification in fascia thickness as older participants showed higher thickness in the lumbar spine than younger participants. Lastly, intramuscular or biological properties of subcutaneous structures may also influence the viscoelasticity characteristics of older participants.

There are limitations in our study that should be considered: (1) We did not record muscle activity during acquisitions. EMG could have been used to make sure that the muscle was inactive (Andonian et al., 2016). However, active muscular activity can be seen by the examiner during data analysis, although it is not sure that every patient stood in the standardized position and that children were as compliant as our adult participants. Participants that provided outlier patterns due to muscular activity were excluded (*n* = 7). (2) Children were classified by chronological age and not by maturation rate. As is known, the growth rate differs from boys and girls, and this could have biased our results, “childhood” must, therefore, be defined with caution. (3) PMAS was assessed in both flexion/extension muscle groups, providing a general non-specific PMAS for the wrist. However, PMAS of the wrist is mostly provided by fingers and thumb muscles (Zonnino and Sergi, 2019). (4) Analysis of the torque and displacement signals was based on the Hill's three-element muscle model (Hill, 1938). As the movement provided was of low amplitude, we assumed that the signals had linear behavior and therefore applied a linear regression model. However, behavior of the wrist complex is not perfectly linear (see Figure 2) and current results may be overestimated. (5) Sports activities were not recorded (due to lack of answers or evasive answers) and professional activities were omitted. Such activities could have impacted the development of PMAS and should have been considered.

## Perspectives

Developing further clinical trials might provide better guidelines concerning neurological disorders such as spasticity. PMAS could also play a major role in the assessment of many musculoskeletal dysfunctions (Blackburn et al., 2004; Pruyn et al., 2014). Quantifying diminished PMAS could be relevant as PMAS may influence proprioceptive acuity (Marinho et al., 2017). EOD methodology has already been established in the lower limb. Detrembleur and Plaghki (Detrembleur and Plaghki, 2000) demonstrated a significant decrease in PMAS in spastic patients before and after baclofen injections.

In surgical practice, EOD might assist the clinician, for example, in tendon lengthening or to measure tremor reduction by means of deep brain stimulation in Parkinson's disease (Shapiro et al., 2007; Kwon et al., 2014; Özyalvaç et al., 2019). Moreover, EOD could be an interesting post-treatment tool to objectify the success or failure of the latter (Bleyenheuft et al., 2008). The viscoelastic models based on stiffness data extracted from the EOD might be considered in modeling soft tissues for virtual and robotic surgery (Famaey and Vander Sloten, 2008).

In conclusion, EOD permits quantifying PMAS reliably and reproducibly. EL and VI stiffness are mostly explained by body size characteristics, like height, body mass, and forearm circumference. Age variable is moderately associated with stiffness. After body size normalization, EL was no longer influenced by the variables measured. On the other hand, VI remained moderately influenced by age and body size. EOD provides normative data in a large sample of healthy participants that can be used as a reference for pathological disorders in neurological or musculoskeletal fields or in surgery.

## Data Availability Statement

The datasets generated for this study are available on request to the corresponding author.

## Ethics Statement

The studies involving human participants were reviewed and approved by Comité d'ethique Hospitalo-Facultaire de l'UCLouvain. Written informed consent to participate in this study was provided by the participants' legal guardian/next of kin.

## Author Contributions

CD, BH, and PM originated this project. CD previously worked in the field. BH developed the device. CD supervised the work while AN, GE, and TL conducted the experiments. AN wrote the manuscript and realized the statistical analysis under the supervision and advice from CD. All authors discussed the results and actively contributed to the final manuscript.

### Conflict of Interest

The authors declare that the research was conducted in the absence of any commercial or financial relationships that could be construed as a potential conflict of interest.
